# Clinical features and therapeutic challenges of psoriatic arthritis coexisting with antisynthetase syndrome: a case report and literature review

**DOI:** 10.3389/fimmu.2025.1626226

**Published:** 2025-08-08

**Authors:** Rui Yan, Dan Ke, Yan Zhang, Li Li, Yanying Liu, Shengguang Li, Xiaomin Liu

**Affiliations:** ^1^ Department of Rheumatology and Immunology, Beijing Shunyi Hospital, Beijing, China; ^2^ Department of Rheumatology and Immunology, Beijing Friendship Hospital, Capital Medical University, Beijing, China; ^3^ Department of Rheumatology and Immunology, Peking University International Hospital, Beijing, China

**Keywords:** psoriatic arthritis, antisynthetase syndrome, JAK inhibitors, TNF inhibitors, case report

## Abstract

**Objective:**

The coexistence of psoriatic arthritis (PsA) with inflammatory myopathies, including antisynthetase syndrome (ASS), is exceptionally rare and presents significant diagnostic and therapeutic challenges. This study reports a case of PsA overlapping with ASS and reviews the literature to analyze clinical features, immunopathogenesis, and treatment strategies.

**Methods:**

A 52-year-old female with a 10-year history of psoriasis developed PsA and later presented with muscle weakness, mechanic’s hands, and interstitial lung disease (ILD). Serological testing revealed anti-Jo-1 and anti-SSA/Ro52 positivity, confirming ASS. We compare our case with 17 previously reported cases of psoriasis or PsA coexisting with inflammatory myopathies, highlighting similarities and differences in clinical presentation and treatment response.

**Results:**

Psoriasis and inflammatory myopathies share immunopathogenic pathways, including the IL-17/IL-23 axis, type I interferons, and TNF-α. Therapies effective for psoriasis/PsA, such as TNF and IL-17 inhibitors, may exacerbate inflammatory myopathies, while JAK inhibitors and corticosteroids appear more effective in managing overlap cases. Our patient achieved sustained remission with baricitinib and low-dose prednisone after multiple treatment adjustments.

**Conclusion:**

PsA and inflammatory myopathies can coexist, requiring careful differentiation and tailored immunomodulatory therapy. Clinicians should recognize overlapping features and optimize treatment to prevent exacerbations. Further research is needed to establish standardized management strategies for this rare overlap syndrome.

## Introduction

Idiopathic inflammatory myopathies (IM) are a group of idiopathic autoimmune disorders characterized by chronic muscle inflammation and weakness. This category includes polymyositis (PM), dermatomyositis (DM), inclusion body myositis, and other subtypes or overlap syndromes. PM and DM are classic IM subtypes – PM involving primarily proximal skeletal muscles, and DM affecting both skin and muscle. These myopathies are relatively rare (for example, DM affects roughly 1–6 per 100,000 adults) (1). Notably, IM can occur in isolation or in association with other autoimmune connective tissue diseases (2). One important overlap subset is anti-synthetase syndrome (ASS), defined by the presence of anti-aminoacyl tRNA synthetase autoantibodies. ASS is characterized by inflammatory myositis accompanied by systemic features such as interstitial lung disease (ILD), non-erosive polyarthritis, Raynaud’s phenomenon, fever, and “mechanic’s hands” (3). Each of these IM subtypes provides a window into autoimmune muscle pathology, and their co-occurrence with other immune-mediated diseases is an area of clinical interest.

Psoriasis, on the other hand, is a common chronic immune-mediated inflammatory disease primarily affecting the skin. It is often accompanied by psoriatic arthritis (PsA) and other systemic comorbidities. Indeed, patients with psoriasis have higher frequencies of concurrent autoimmune disorders such as rheumatoid arthritis, systemic lupus erythematosus (SLE), systemic sclerosis, Sjögren’s syndrome, and others (4). However, the overlap of psoriasis with idiopathic inflammatory myopathies is exceedingly rare. Co-existence of psoriasis and DM has been documented only in a handful of cases in the literature. A recent review identified roughly 15 reported instances of psoriasis co-occurring with dermatomyositis (including some juvenile and amyopathic DM cases) (5). These observations underscore the rarity of psoriasis–myositis overlap, in contrast to the well-recognized psoriasis–PsA association.

The uncommon overlap between psoriasis and IM subtypes raises intriguing questions about shared pathogenesis and immune crosstalk. Some authors have speculated that common inflammatory pathways might link these conditions. Dermatomyositis, for instance, features a prominent type I interferon signature and involvement of plasmacytoid dendritic cells, immunologic elements also central in psoriasis (4). Furthermore, elevated levels of interleukin-17 (IL-17) have been observed in DM patients, and IL-17–producing T cells infiltrate the lesions of both psoriasis and DM, suggesting a contribution of the IL-23/Th17 axis to both diseases (6). Clinical reports lend support to this mechanistic overlap – notably, therapies targeting the IL-23/IL-17 pathway (e.g. ustekinumab, an IL-12/23 p40 inhibitor used in psoriasis) have shown efficacy in treating refractory “mechanic’s hands” in an antisynthetase syndrome patient (7). Such findings hint at converging immunopathological drivers despite the rarity of co-manifestation.

Antisynthetase syndrome itself represents an illustrative example of overlap between an inflammatory myopathy and features that can mimic other rheumatic diseases. Patients with ASS often develop an inflammatory polyarthritis as part of the syndrome, which in a psoriasis patient could be initially mistaken for psoriatic arthritis. Indeed, at least one case report describes a patient with longstanding psoriasis/PsA who subsequently developed dermatomyositis with anti-Jo-1 antibodies (fulfilling ASS), creating a diagnostic challenge of distinguishing PsA from myositis-related arthritis (8). In this paper, we highlight the overlap of psoriasis (and psoriatic arthritis) with an antisynthetase syndrome, situating it within the broader spectrum of psoriatic disease coexisting with inflammatory myopathies. Our aim is to enhance recognition of this uncommon association and discuss its implications for pathogenesis and patient management.

## Case presentation

A 52-year-old female teacher was admitted in June 2019 due to a 10-year history of scaly rash and a recent 1-month history of polyarticular swelling and pain. Her initial skin symptoms appeared a decade prior as scaly rash involving her limbs, gradually spreading across her entire body, accompanied by pinpoint bleeding upon scratching. Initially diagnosed as diffuse plaque psoriasis, her skin lesions showed fluctuating severity despite intermittent topical treatment. She had no relevant psoriasis family history.

Approximately six months prior to hospitalization, she began experiencing mild exertional dyspnea and occasional dry cough, untreated at that time. One month before admission, she developed persistent joint pain and swelling involving bilateral metacarpophalangeal joints (MCPJ), proximal interphalangeal joints (PIPJ), knees, and ankles, significantly impairing mobility. Occasional lower back pain was noted, and her lower back pain had inflammatory characteristics. The patient had no Raynaud’s phenomenon, with no dryness of mouth or eyes, recurrent oral ulcers, or alopecia. She had no history of hypertension or diabetes mellitus.

**Figure 1:**
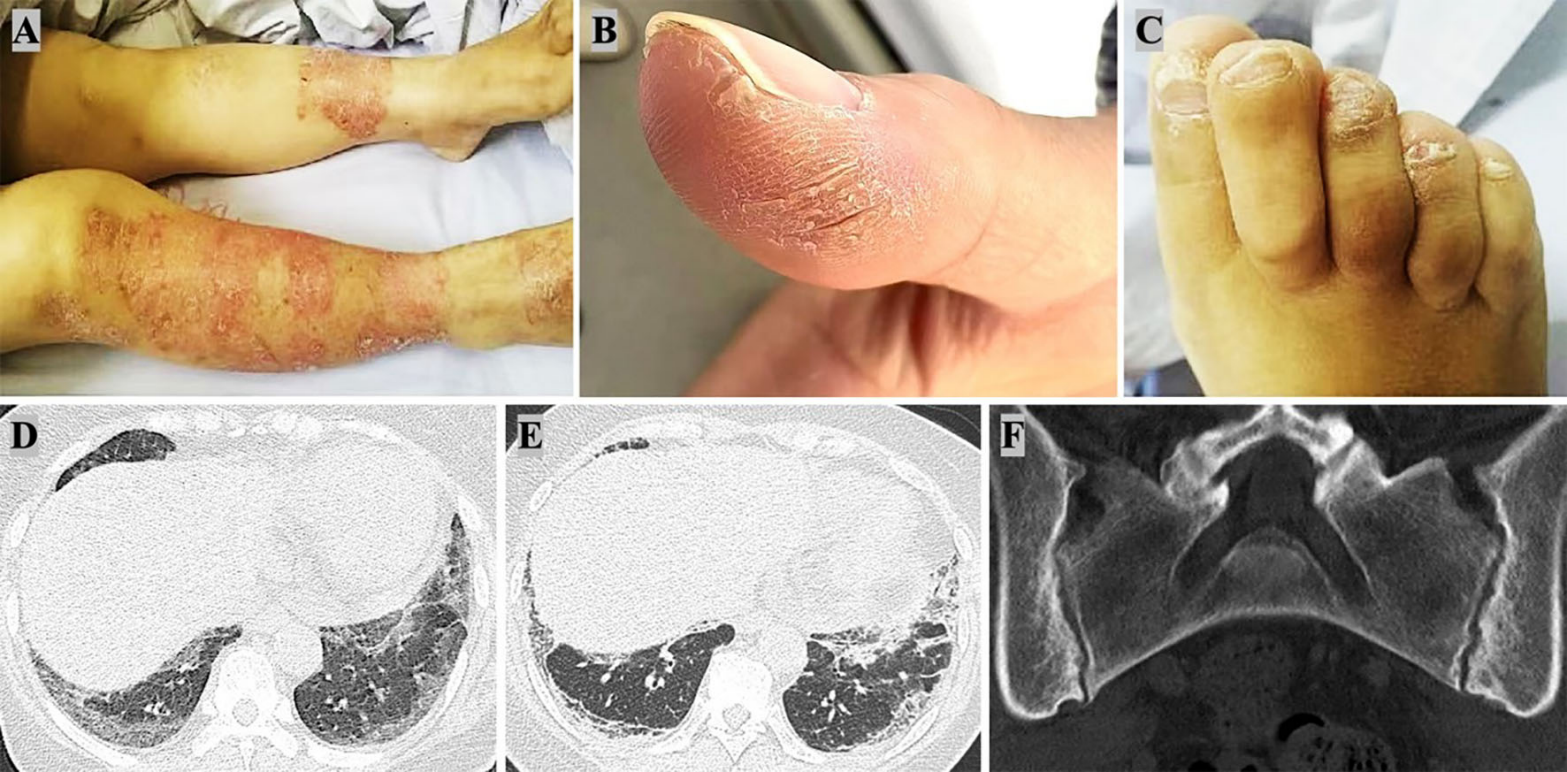
Clinical and Radiological Manifestations of Antisynthetase Syndrome in a Patient with Psoriatic Arthritis. **(A)** Scaly, erythematous plaques with well-demarcated borders distributed on the lower extremities, characteristic of psoriasis. **(B)** Hyperkeratosis and fissuring of the thumb, consistent with mechanic’s hands, a hallmark feature of antisynthetase syndrome. **(C)** Thickened, dystrophic toenails with onychodystrophy and dactylitis, commonly seen in psoriatic arthritis (PsA). **(D)** High-resolution computed tomography (HRCT) scans showing interstitial lung disease with a nonspecific interstitial pneumonia (NSIP) pattern, which is frequently associated with antisynthetase syndrome (on admission). **(E)** Interstitial lung disease exacerbated because of inadequate treatment. **(F)** Computed tomography (CT) scan of the sacroiliac joints showing mild bilateral sacroiliitis, which can occur in psoriatic arthritis.

On physical examination, vital signs were stable. Scaly plaques were observed on the dorsal hands and lower extremities (**Figure 1A**), with scattered dandruff on the scalp. Psoriasis Area and Severity Index (PASI) was 3.9. Bilateral basal lung crackles were audible. Cardiac and abdominal examinations were unremarkable. Both hands displayed mechanic’s hands appearance (**Figure 1B**), with swelling and tenderness noted at the left PIP2–3 joints and the right knee. The second toe of the left foot and the fourth toe of the right foot exhibited dactylitis, showing a “sausage toe” appearance (**Figure 1C**). Partial nail thickening and whitening were observed (**Figure 1C**).

Routine laboratory investigations were normal, including complete blood count, liver and kidney functions. Autoimmune tests revealed positivity for anti-Jo-1 and anti-SSA-Ro-52 antibodies, with elevated KL-6 levels (983 U/mL; normal 105–435 U/mL). HLA-B27 was negative. Radiographs of hands and knees showed no erosions, whereas chest CT demonstrated interstitial changes consistent with a nonspecific interstitial pneumonia (NSIP) pattern (**Figure 1D**). Pulmonary function tests revealed restrictive ventilatory impairment with reduced diffusion capacity (DLCO: 60% predicted). Sacroiliac joint CT indicated mild erosion (**Figure 1F**).

**Figure 2:**
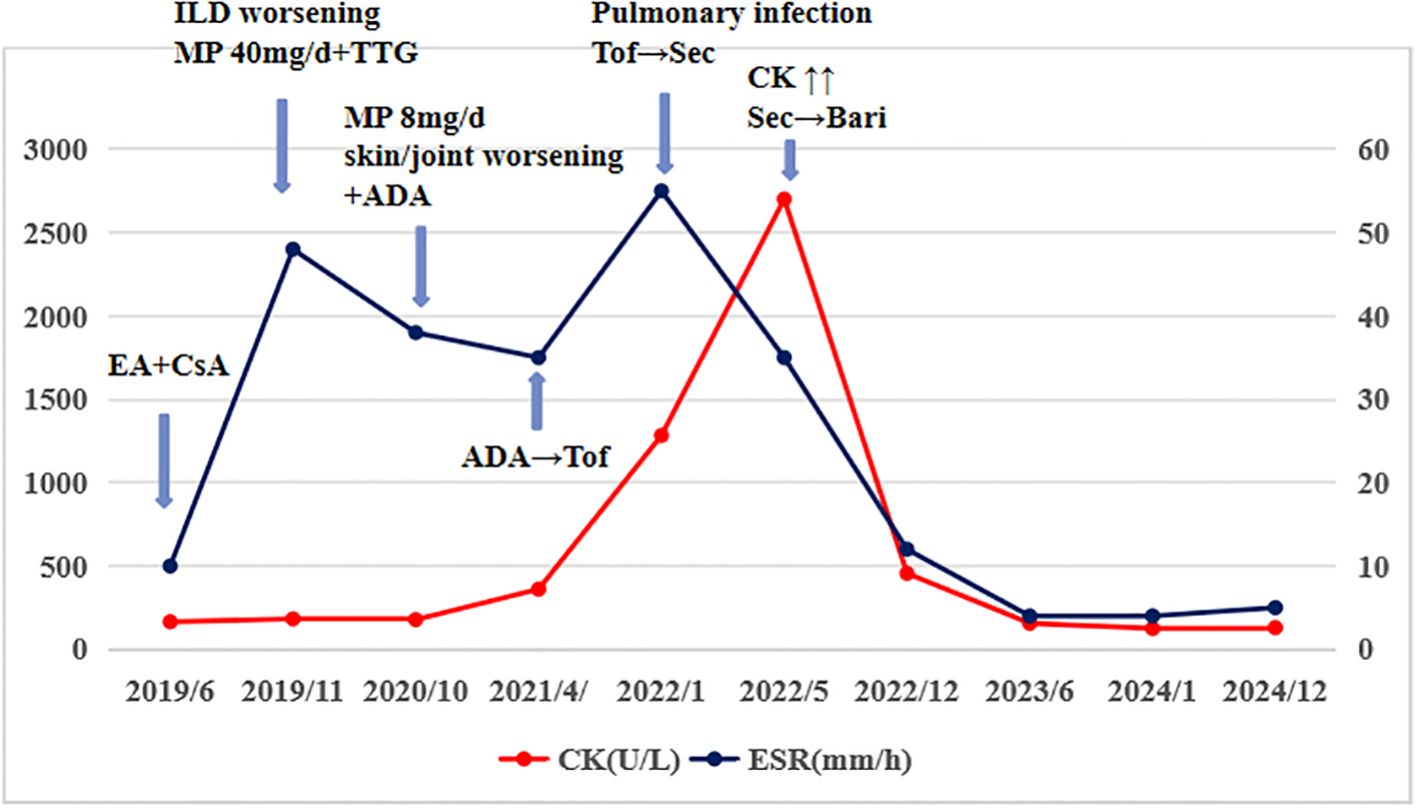
The management flowchart for this patient. EA, etanercept analog, CsA, cyclosporine; ILD, intersitial lung disease; MP, methylprednisolone, TTG, tripterygium glycosides; ADA, adalimumab; Tof, tofacitinib; Sec, secukinumab; CK↑↑, creatine kinase elevated significantly; Bari, baricitinib; ESR, erythrocyte sedimentation rate.

The patient was diagnosed with PsA according to CASPAR criteria and ASS based on Connors criteria. Initial treatment for PsA included a weekly subcutaneous injection of etanercept analog (Yisaipu) 50mg and oral cyclosporine 100mg twice daily for ILD. Due to gastrointestinal intolerance of cyclosporine, she was switched to tacrolimus 1mg twice daily and improved skin and joint symptoms but worsened ILD (**Figure 1E**). Treatment was then adjusted to methylprednisolone and tripterygium glycosides, stabilizing disease. Tapering the glucocorticoid led to worsening skin and joint symptoms. Later, ineffective treatment with adalimumab and subsequent tofacitinib were complicated by pulmonary infection and elevated creatine kinase (CK, 361 U/L). Although secukinumab transiently improved symptoms, CK markedly elevated (2701 U/L) after one year, indicating drug-induced enzyme elevation. Finally, switching to baricitinib (4 mg/day) combined with methylprednisolone (8 mg/day) achieved sustained remission of psoriasis, arthritis, and normalization of CK levels over two years. **Figure 2** shows the management flowchart for this patient.

## Methods

We systematically searched the PubMed and Embase databases up to May 2025, using the keywords ‘psoriasis’, ‘psoriatic arthritis’, ‘antisynthetase syndrome’, ‘dermatomyositis’, and ‘inflammatory myopathy’. Inclusion criteria were clearly documented cases of psoriasis or PsA overlapping with inflammatory myopathies. Exclusion criteria included incomplete clinical data or unclear diagnoses.

## Discussion

To date, a comprehensive search has identified only 17 previously reported cases of psoriasis or psoriatic arthritis coexisting with inflammatory myopathies where clinical details were sufficiently reported, thus our case represents the 18^th^ documented instance **Table 1** summarizes the key clinical characteristics of these cases (2, 5, 8–20). Our patient – to our knowledge is one of the first reported with psoriatic arthritis (PsA) overlapping with anti-Jo1 antisynthetase syndrome (8). This rarity highlights the importance of recognizing the overlap syndrome, as misdiagnosis or delayed diagnosis can lead to suboptimal treatment outcomes. The coexistence of psoriasis and myositis presents unique diagnostic and therapeutic challenges due to overlapping symptomatology and differences in treatment responses. Understanding the shared and distinct immunopathogenic mechanisms between these diseases is critical for optimal patient management.

**Table 1:** Clinical features and treatment of psoriasis with inflammatory myopathies.

Author/(Ref)	Year published	Gender	Age (year)	Age of IM Onset	Age of Psoriasis Onset	Myositis type	Preciasis pariatic arthritis type	Triggers for disease	Autoantibody	Treatment	Response
Pastivic MD, etal./ (9)	2004	Male	63	59	63	DM	Psoriasis	Unknown	NA	Topical steroids, keratolyties, topical calcipotriol	Good response, lesions cleared stable remission
Machado NP, et al./ (10)	2010	Male	51	41	45	DM	Psoriasis	Unknown	ANA+, pANCA+	High-dose steroids, cyclophosphamide	Partial renal remission with ryclophosphamide
Kim NN, et al. (Case 1)/ (11)	2011	Female	8	8	18	JDM	Psoriasis	Unknown	NA	IV methylprednidolone, oral predniscone, methotorsate	Good response initially to steroids partial improvement with methotresate
Kim NN, et al. (Case 2)/ (11)	2011	Female	7	8	7	JDM	PsA	Etanercept triggered DM	NA	IV methylprednisolone, oral prednisone, methotrexate, mycophenolate modetil, cyclosporine, topical steroids;	Initial proviasis improvement; JDM worsened with etanement, improved with steroids, methotresate, mycophenolate mofetil
Kim NN, et al. (Case 3)/ (11)	2011	Female	4	2	4	JDM	Psoriasis	Steroid tapering triggered psoriasis	NA	High-dose IV and low-dose oral steroids, topical corticosteroids	Good response; symptom free after treatment cessation
Dicaro D, et al./ (12)	2014	Female	37	37	30	ADM	Psoriasis	Adalimunab triggered ADM	negative	Methotresate, cessation of adalimumab	Partial improvement
Akiyama M, et al./ (13)	2016	Female	52	44	50	DM	Psoriasis	Withdrawal of prednsolone triggered psoriasis	ANA+ SSA+ TIF-1+	Preduisclone, Methotresate	Good symptoms controlled
Kato Y, et al./ (14).	2017	Female	30	Before 30	Before 30	DM	Psoriasis	Unknown	ANA+	Initial: methyfprednisolone pulse; Later: increased oral	Successful skin lesion resolution after increased dose of prednisolone
Inkeles MS, et al./ (15)	2017	Female	45	41	Before 41	ADM	Psoriasis	Unknown	ANA+	Cyclosporine (5 mg/kg/day)	Good response after multiple failed treatments restained remission
Montoya CL, et al./ (16).	2017	Male	20	15	20	AJDM	Erythrodemic Psoriasis	Rituximab triggmed psoriasis	Negative	Ustekimanab	Excellent
Sofi F, et al./ (2)	2018	Male	35	35	34	PM	Psoriasis	Unknown	NA	Prednisone, Azathioprine	Good response for psoriasis, after treated with prednisone, PM remission after adding azathioprine
Xing Y, et al./ (17)	2019	Male	21	15	21	JDM	Psoriasis	Unknown	NA	Corticosteroid, cyclosporin methotrexate, topical calcipotriol and corticosteroids, antifungals	Good
Schreiber C, et al./ (18)	2021	Female	45	45	Pre-existing PsA	DM	PsA	Unknown	Anti Jo+ AMA+ ASMA+	Prednisone, Azathioprine, Mycophenolic acid Methotrexate	Initial steroid response, relapse, partial control with methotrexate
Hanami Y, et al./ (8)	2021	Female	74	Unknown	64	ASS	Psoriasis	Unknown	ANA+,ARS+	Not mentioned	Not mentioned
Perma DL, et al./ (19).	2022	Female	20s	Juvenile onset	Juenile onset	JDM	Psoriasis	Secukinumab triggered DM	Negative	Discontinuation of Secukinumb methotrexate reintroduced	DM improved after discontinuation but flared upon rechallenge
Chu D, et al./ (5)	2024	Male	63	63	53	DM	Psoriasis	Sunlight exposure triggered psoriasis	Negative	Methylprednisolone, hydroxychloroquine,	Significant improvement
Xu Z, et al./ (20)	2024	Female	41	41	14	DM	Psoriasis	Adalimunab triggered DM	ANA+	Adalimunab discontinued, Betamethasone ineffective, Upadacitinib (JAK1 inhibitor)	Significant improvement is skin lesions and nuscle symptoms after Upadacitinib
Present case	2025	Female	52	51	42	ASS	PsA	Unknown	ANA+, Asti-Jo-1+	Etanercept, Carrolinnas, Methylprednisolone, Tripterygium, Tofacitinib, Secukinmab baricitinib	Partial initial improvement, disease exacerbation, finally controlled by Secukimmab and subsequently stable remission with baricitinib

This table presents a comparison between our case of psoriatic arthritis (PsA) with antisynthetase syndrome (ASS) and 17 previously reported cases of psoriasis or psoriatic arthritis coexisting with various inflammatory myopathies (including dermatomyositis (DM), polymyositis (PM), and juvenile dermatomyositis (JDM)). It summarizes patient demographics, clinical characteristics, triggers for disease onset, autoantibody profiles, treatment strategies, and responses. This comprehensive comparison highlights commonalities and differences in the presentation and management of this rare overlap syndrome, providing valuable insights for clinicians in managing similar cases.

(ASS, antisynthetase syndrome; DM, dermatomyositis; JDM, juvenile DM; CADM, clinically amyopathic DM; PsA, psoriatic arthritis; ILD, interstitial lung disease; ARS, anti-aminoacyl-transfer RNA synthetase antibody; NA, Not available; MTX, methotrexate; PSL, prednisone/prednisolone; TNFi, TNF inhibitor; IL-17i, IL-17 inhibitor; JAKi, JAK inhibitor).

Despite being distinct disorders, psoriasis/PsA and inflammatory myopathies share certain inflammatory pathways, which may explain why they rarely coexist. T helper 17 (Th17)/IL-23 axis: Psoriasis is driven largely by IL-23–induced Th17 cells producing IL-17A/F and other cytokines (21, 22). Notably, this same pathway appears active in myositis. Patients with DM/PM have elevated IL-17A levels in serum correlating with disease activity (23), and muscle biopsies show higher IL-17 and IL-23 expression compared to controls (24). An experimental myositis model demonstrated that blocking IL-23 (with anti-IL-23p19 antibodies) suppressed muscle inflammation, highlighting IL-23 as a pathogenic driver in myositis much like in psoriasis (24). Tumor necrosis factor-α: TNF-α is a proinflammatory cytokine central to psoriasis/PsA and also found in inflamed muscle tissue. Muscle from DM/PM patients’ over-expresses TNF-α along with IL-17 and IFN-γ, reflecting broad T-cell activation (25). Anti–TNF therapies (e.g. etanercept, adalimumab) are highly effective for psoriasis, and their use in our case initially helped control skin and joint symptoms. However, TNF inhibition has paradoxically been linked to new-onset or exacerbation of myositis in predisposed patients (25). Indeed, psoriasis patients exposed to TNF blockers in one series had a significantly higher odds of developing myopathy (OR ~4.45) (26), suggesting TNF may also have complex immunoregulatory roles. Type I interferons: DM, especially the cutaneous form, is strongly associated with a type I interferon gene signature. In contrast, psoriasis is classically a *Th17/Th1* disease, but type I IFN can act as an upstream trigger – plasmacytoid dendritic cells infiltrating psoriatic skin produce IFN-α that helps initiate psoriatic plaques (27). We specifically highlight the immunopathogenesis of ASS, including anti-aminoacyl-tRNA synthetase antibodies, abnormal activation of autoreactive B cells, and type I interferon-driven immune response, clearly differentiating from dermatomyositis mechanisms. Thus, both conditions exhibit *innate and adaptive immune interplay*: IFN-α and TNF-α from innate immune cells drive activation of autoreactive T-cells (Th17, Th1), which then release IL-17, IFN-γ, TNF and other mediators. The overlapping cytokine profile (e.g. IL-6, IL-17, TNF, IFN) provides a mechanistic basis for why treating one disease can affect the other. It also suggests a genetic or immunologic predisposition in rare patients who develop both diseases. Notably, no shared HLA risk allele has been identified (psoriasis is tied to HLA-C*06:02/B*27, whereas antisynthetase/DM have links to HLA-DRB1*03/*08) (28), implying that the overlap arises from convergent inflammatory pathways rather than a common genetic cause. A Retrospective Cohort Study demonstrated that psoriatic patients with PsA or those who had received IL-17i treatment demonstrated a significantly higher risk of developing dermatomyositis (29).

Because psoriasis/PsA and inflammatory myopathies can produce overlapping clinical features, patients with both are at high risk of misdiagnosis. Skin manifestations of dermatomyositis may be mistaken for psoriasis, and vice versa. For example, DM classically causes erythematous scaly papules over extensor joints (Gottron’s papules), which could be confused with psoriatic plaques on the elbows, knees, or knuckles. In one reported case, a 41-year-old woman developed scaly erythematous lesions on her hands, elbows and knees and symmetric polyarthritis; this was initially diagnosed and treated as psoriatic arthritis, only to later be recognized as anti-MDA5 positive dermatomyositis when muscle weakness, rash distribution, and specific antibodies declared themselves (30). Our patient’s presentation also illustrates the potential for diagnostic confusion. She had a ten-year history of psoriasis and nail changes, then developed polyarthritis with dactylitic “sausage” toes and even sacroiliitis on imaging – all classic PsA features. However, concurrently she exhibited mechanic’s hands (hyperkeratotic fissuring of the fingertips) and interstitial lung disease (bibasilar fibrosis on CT with reduced diffusion capacity). Mechanic’s hands and unexplained ILD should raise suspicion for antisynthetase syndrome, but in a patient already known to have psoriatic disease, these findings could easily be attributed to psoriasis (e.g. mistaken as severe hand psoriasis or a drug-induced lung reaction). The key to diagnosis is recognizing features that cannot be explained by psoriasis/PsA alone. In our case, the presence of high-titer anti-Jo-1 antibodies, together with mechanic’s hands and ILD, was a red flag that psoriatic disease alone was not the full story. Indeed, anti-Jo-1 is specific for antisynthetase syndrome and is not associated with psoriasis. Thus, a careful history, thorough physical exam, and autoimmune serologies were crucial to establish the overlap diagnosis.

From a rheumatologic perspective, inflammatory arthritis in antisynthetase syndrome can mimic psoriatic arthritis. Antisynthetase patients often have a symmetric polyarthritis (sometimes erosive) that could be misdiagnosed as rheumatoid or psoriatic arthritis if the other hallmarks of myositis are subtle. Similarly, cutaneous signs like mechanic’s hands can resemble acral psoriasis or chronic hand dermatitis (31). It is noteworthy that antisynthetase syndrome has a range of cutaneous findings (rough, cracked skin on fingers, “dirty” hyperpigmented lesions) that dermatologists caution can look like more common conditions including psoriasis. Muscle symptoms may also be overlooked in the context of psoriatic disease. Patients with PsA frequently report fatigue and diffuse pain, which might mask or delay recognition of true muscle weakness. In our patient, creatine kinase was only mildly elevated at times despite active myositis, making muscle involvement less obvious. Such cases underscore the need for vigilance: clinicians should reassess any psoriatic patient who develops atypical features (unexplained ILD, proximal muscle weakness, distinct rashes) and consider screening with muscle enzymes or autoantibody panels. Conversely, in DM patients with chronic rash, a psoriatic component should be considered if lesions have the classic silver scale or if arthritis and nail dystrophy are present. In summary, diagnosing this overlap requires a high index of suspicion and often a multidisciplinary approach (dermatology, rheumatology, pulmonology) to piece together the two coexisting conditions.

Managing a patient with both psoriasis/PsA and an inflammatory myopathy is challenging, as therapies must be chosen to control both diseases without provoking the other. There are no established guidelines for this rare overlap, so treatment is largely individualized. Our case vividly demonstrated that a trial-and-error approach may be needed before finding a regimen that balances both conditions. Key therapeutic considerations include the following:

- Tumor Necrosis Factor (TNF) Inhibitors: TNF blockers (etanercept, adalimumab, etc.) are highly effective for moderate-severe psoriasis and PsA, and they were used early in our patient’s course. In the short term, etanercept helped our patient’s skin and joint symptoms. However, TNF inhibition does not treat myositis and may even worsen certain aspects. Our patient’s interstitial lung disease progressed while on etanercept, possibly because her antisynthetase syndrome was insufficiently controlled. There is also evidence that TNF inhibitors can trigger or unmask myositis in predisposed individuals: several reports detail new-onset dermatomyositis occurring after 2–6 months of etanercept or adalimumab therapy for arthritis (32). In one series of psoriasis patients, those exposed to TNF inhibitors had a significantly higher likelihood of developing inflammatory myopathy than those never treated with biologics (26). Although a causal relationship remains controversial, the implication is that TNF has a complex role – its blockade can precipitate autoimmunity (e.g. lupus-like syndromes, vasculitis, and rarely dermatomyositis) (32). We clarify that while TNF inhibitors have been associated with myositis onset or exacerbation, this remains an association rather than definitive causation. Clinicians should remain vigilant when using TNF inhibitors in patients predisposed to inflammatory myopathies.
- IL-17 and IL-23 Inhibitors: Agents targeting the IL-17/IL-23 pathway are highly effective for psoriasis and PsA, but caution is warranted in the setting of myositis. There have been reports of dermatomyositis onset or exacerbation temporally linked to IL-17 blockade. For example, a recent report described a psoriasis patient who experienced severe DM flares associated with secukinumab (IL-17A inhibitor) treatment (19). In our patient, after other therapies failed, we initiated secukinumab to control her psoriatic disease. This achieved partial improvement of skin and joint symptoms, but we observed a significant rise in muscle enzymes (CK climbed to >1200 U/L) despite the patient feeling no immediate weakness. This subclinical CK elevation suggested that IL-17 inhibition was aggravating her myositis. After 1 year on secukinumab, her CK had spiked further (>2700 U/L), prompting us to discontinue the IL-17 inhibitor. IL-23 p19 inhibitors (e.g. guselkumab) have not been reported in this overlap to our knowledge, but IL-12/23 inhibition with ustekinumab has been tried. Notably, one case of juvenile DM with psoriasis was successfully treated with ustekinumab, achieving remission of both skin and muscle inflammation (16). This aligns with mechanistic data that IL-23 is a driver of myositis as well as psoriasis (24). Due to the limited available data, the therapeutic role of newer biologics such as IL-23 inhibitors in PsA-ASS overlap remains unclear. Future studies specifically addressing this gap are warranted to clarify their clinical efficacy and safety.
- JAK Inhibitors: Janus kinase inhibitors (tofacitinib, baricitinib, upadacitinib, etc.) have emerged as an appealing option for overlapping psoriasis and myositis. These oral small molecules broadly modulate cytokine signaling (blocking pathways of IFN, IL-6, IL-12/23, etc.), which means they can potentially treat both Th17-mediated skin disease and interferon-mediated muscle disease simultaneously. Our patient’s most sustained remission was achieved with the JAK1/2 inhibitor baricitinib (4 mg daily) combined with low dose prednisone. Over 12 months, this regimen led to resolution of her arthritis and psoriatic lesions and normalization of CK levels, indicating control of the myositis. JAK inhibitors have shown efficacy in refractory dermatomyositis in recent studies, especially for patients with prominent interferon signatures (e.g. anti-MDA5 DM) – likely by inhibiting type I IFN signaling. In psoriasis and PsA, tofacitinib (a JAK3/1 inhibitor) is an approved therapy that improves both skin and joints. In one reported overlap case, a patient with severe psoriasis who developed DM after adalimumab was switched to the JAK1 inhibitor upadacitinib, with significant improvement in both diseases (5). Our patient similarly experienced improvement when we tried tofacitinib, though an intercurrent infection led us to change course at that time.
- Corticosteroids and Conventional Immunosuppressants: Systemic corticosteroids remain the first-line therapy for idiopathic inflammatory myopathies and were indispensable in our patient’s regimen. High-dose prednisone or intravenous methylprednisolone often yields rapid improvement in muscle strength and lung function in myositis (15). In our case, prednisone 40 mg daily stabilized the ILD and muscle disease when other drugs were withdrawn. The dilemma is that long-term steroids can worsen psoriasis and PsA (and steroid tapering may trigger psoriasis rebound or pustular flares). We managed this by maintaining a moderate steroid dose until other steroid-sparing agents took effect, and fortunately our patient did not experience a psoriatic flare upon taper. DMARDs: Traditional immunosuppressants like methotrexate (MTX), azathioprine, cyclosporine, and mycophenolate are commonly used as steroid-sparing agents in both psoriasis/PsA and myositis. MTX, for instance, can control PsA and has modest efficacy in myositis, though data are limited. Several reported overlap cases were treated successfully with methotrexate or azathioprine plus prednison (5). In our patient, we attempted cyclosporine and later tacrolimus (calcineurin inhibitors) alongside biologics; however, she did not tolerate cyclosporine and tacrolimus had to be stopped due to ILD progression. Ultimately, unconventional therapies like Tripterygium wilfordii (a Chinese herbal immunosuppressant) were also employed in our case to help control disease activity.

In conclusion, the coexistence of psoriasis/PsA with inflammatory myopathies like DM, PM, or antisynthetase syndrome, while rare, is an important clinical phenomenon that demands careful attention. Recognizing the overlap is clinically significant – it prompts comprehensive evaluation (including for ILD and autoantibodies) and a tailored management strategy. Shared immunopathogenic mechanisms, particularly the IL-17/IL-23 axis and other inflammatory pathways, likely underlie the link between these conditions, and targeting these pathways can be a double-edged sword. Diagnostic vigilance is required to distinguish overlapping features and avoid misdiagnosis. Management must be holistic and often multi-disciplinary, balancing therapies to control both muscle and skin/joint disease. Our case, placed in context with the literature, highlights both the challenges and potential solutions in treating this uncommon overlap syndrome. We acknowledge that conclusions drawn from the present single case and small number of previously reported cases should be interpreted with caution. Individualized treatment strategies remain essential in managing this rare overlap syndrome. As more cases are reported and studied, we will better elucidate the connections between these diseases and optimize care for patients who unfortunately encounter both.

## Data Availability

The original contributions presented in the study are included in the article/supplementary material. Further inquiries can be directed to the corresponding authors.
